# Supplementary material for: MetaRibo-Seq measures translation in microbiomes
Source: Nat Commun. 2020 Jun 29;11:3268. doi: 10.1038/s41467-020-17081-z (PMC7324362; doi:10.1038/s41467-020-17081-z)
Supplement: Supplementary file 10 — Supplementary Data 7 [file 41467_2020_17081_MOESM10_ESM.zip › File2/Confidence_VeryHigh_Taxonomy/57691_out.krona.html]

Javascript must be enabled to view this page.

members
magnitude
magnitudeUnassigned
count
unassigned
taxon
rank

57691\_out

7

7
superkingdom
2

1239
phylum
7

5
class
909932

5
order
909929

1843491
family
5

genus
1
82202

species
1

SRS044448\_contig\_number\_contig-100\_9909.231681
82203

4
genus
970


SRS014402\_contig\_number\_21102SRS014578\_contig\_number\_6304SRS101445\_contig\_number\_49385
135082
3
species

1884263

SRS011152\_contig\_number\_contig-100\_23680.50989
1
species

186801
2
class

186802
2
order

1
family
31979

1485
genus
1

1
species
1650661

SRS147977\_contig\_number\_contig-100\_12732.12733

family
1
216572

459786
genus
1

species
1
1897011

SRS1041095\_contig\_number\_contig-100\_21686.21687
